# *catena*-Poly[[bis­(nitrato-κ*O*)cadmium]bis­[μ-1,3-bis­[(1*H*-1,2,4-triazol-1-yl)meth­yl]benzene-κ^2^ *N* ^4^:*N* ^4′^]]

**DOI:** 10.1107/S1600536812023288

**Published:** 2012-05-31

**Authors:** Hong-Kun Zhang, Xin Wang, Shuai Wang, Xiao-Dan Wang

**Affiliations:** a Department of Food and Environmental Engineering, Heilongjiang East University, Harbin 150086, People’s Republic of China; b College of Chemistry and Materials Science, Heilongjiang University, Harbin 150080, People’s Republic of China

## Abstract

In the title compound, [Cd(NO_3_)_2_(C_12_H_12_N_6_)_2_]_*n*_, the Cd^II^ cation is located on an inversion center and is six-coordinated by four N atoms from four 1,3-bis­[(1*H*-1,2,4-triazol-1-yl)meth­yl]benzene (*L*) ligands and two O atoms from two nitrate anions in a slightly distorted octa­hedral geometry. The ligands link different Cd^II^ ions into a ribbon-like structure along [001]. Two O atoms of the nitrate anion are disordered over two sets of sites with site occupancies of 0.575 (8) and 0.425 (8).

## Related literature

For related structures, see: Meng *et al.* (2004 ▶). For the synthesis of the ligand, see: Du *et al.* (2008 ▶).
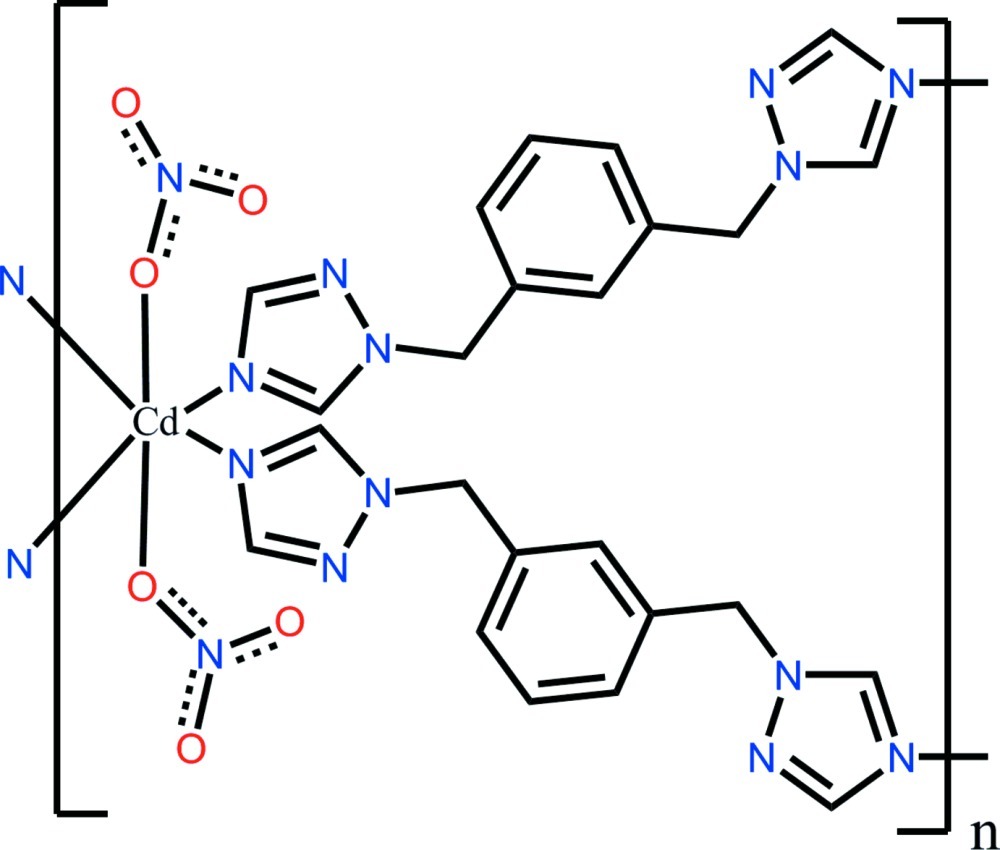


## Experimental

### 

#### Crystal data

[Cd(NO_3_)_2_(C_12_H_12_N_6_)_2_]
*M* *_r_* = 716.97
Triclinic, 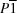

*a* = 8.0412 (16) Å
*b* = 8.7303 (17) Å
*c* = 11.598 (2) Å
α = 105.12 (3)°
β = 90.20 (3)°
γ = 109.71 (3)°
*V* = 736.2 (2) Å^3^
*Z* = 1
Mo *K*α radiation
μ = 0.81 mm^−1^
*T* = 293 K
0.41 × 0.28 × 0.15 mm

#### Data collection

Rigaku R-AXIS RAPID diffractometer
Absorption correction: multi-scan (*ABSCOR*; Higashi, 1995 ▶) *T*_min_ = 0.734, *T*_max_ = 0.886
7285 measured reflections
3346 independent reflections
3216 reflections with *I* > 2σ(*I*)
*R*_int_ = 0.020

#### Refinement

*R*[*F* ^2^ > 2σ(*F* ^2^)] = 0.030
*wR*(*F* ^2^) = 0.080
*S* = 1.03
3346 reflections
224 parameters
3 restraints
H-atom parameters constrained
Δρ_max_ = 0.77 e Å^−3^
Δρ_min_ = −0.68 e Å^−3^

### 

Data collection: *RAPID-AUTO* (Rigaku, 1998 ▶); cell refinement: *RAPID-AUTO*; data reduction: *CrystalClear* (Rigaku/MSC, 2002 ▶); program(s) used to solve structure: *SHELXS97* (Sheldrick, 2008 ▶); program(s) used to refine structure: *SHELXL97* (Sheldrick, 2008 ▶); molecular graphics: *SHELXTL* (Sheldrick, 2008 ▶); software used to prepare material for publication: *SHELXL97*.

## Supplementary Material

Crystal structure: contains datablock(s) I, global. DOI: 10.1107/S1600536812023288/vn2040sup1.cif

Structure factors: contains datablock(s) I. DOI: 10.1107/S1600536812023288/vn2040Isup2.hkl

Additional supplementary materials:  crystallographic information; 3D view; checkCIF report

## Figures and Tables

**Table 1:** Selected bond lengths (Å)

Cd1—N1	2.330 (2)
Cd1—N4^i^	2.3278 (19)
Cd1—O1	2.479 (5)

Symmetry code: (i) 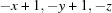.

**Fig. 1.**
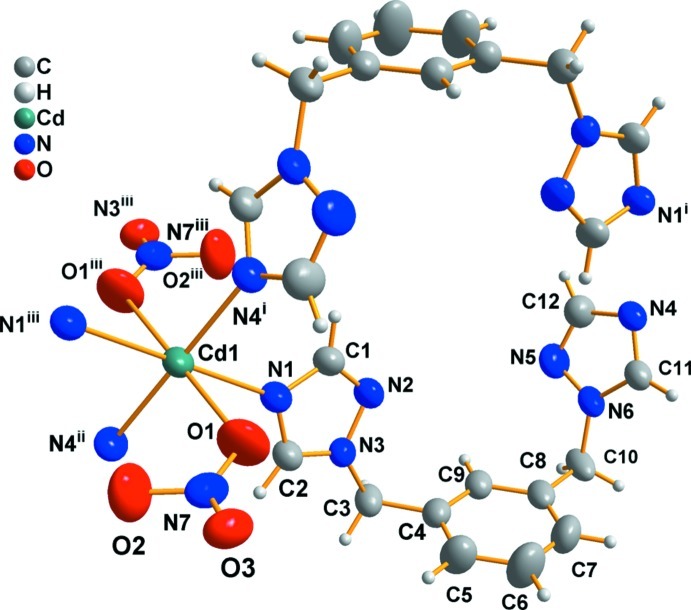
The molecular structure of the title compound, showing displacement ellipsoids at the 50% probability level for non-H atoms. Only the main component of the disordered nitrate anion is shown. Symmetry codes: (i) 1-x, 1-y, 1-z; (ii) 1-x, 1-y, -z; (iii) x, y, 1+z.

**Fig. 2.**
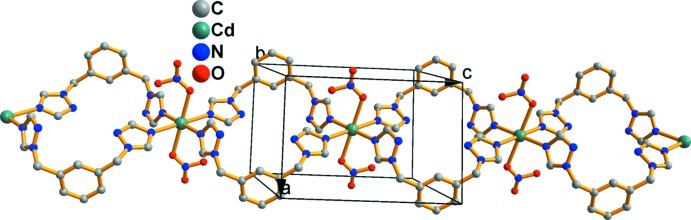
A portion of the ribbon-like structure in the title compound. H atoms and disordered O atoms have been omitted for clarity.

## supplementary crystallographic information

### Comment

In recent years, much attention has been paid to the use of nitrogen-containing ligands for constructing supramolecular coordination compounds. The reason is that the supramolecular coordination assemblies have not only a variety of architectures but also have potential applications as functional materials. Recently, a series of supramolcular complexes based on the 1,4-bis(1*H*-1,2,4-triazol-1-yl-methyl)-benzene ligand were reported (Meng *et al.*, 2004). In this paper, we report the new title compound, synthesized by the reaction of 1,3-bis((1*H*-1,2,4-triazol-1-yl)methyl)benzene and cadmium dinitrate in an aqueous solution.

In the title compound, [Cd(NO3)_2_(C_12_H_12_N_6_)_2_]_n_, the eight-coordinated Cd^II^ ion is located on an inversion center and is in an octahedral environment defined by four N atoms from ligands forming the equatorial plane (distances Cd—N1 = 2.330 (2) Å and Cd—N4 = 2.328 (2) Å) and two O atoms from two nitrate anions lying on the polar axis with a Cd—O1 distance of 2.479 (5) Å (Figure 1, Table 1). An infinite ribbon-like structure running along [001] is built up by the *cis*-ligands linking these Cd^II^ ions (Figure 2).

### Experimental

The ligand *L* was synthesized following the reference method (Du *et al.*,2008). Synthesis of the title compound: *L* (0.120 g, 0.5 mmol) and Cd(NO3)_2_ (0.152 g, 0.5 mmol) were dissolved in a mixed solution of 3 mL ethanol and 3 mL water. After stirring, the suspension was sealed in a 18 mL Teflon-lined autoclave and heated at 140 °C for 5 days. After slow cooling to room temperature, colorless block crystals were filtered and washed with distilled water (47% yield based on Cd).

### Refinement

H atoms bound to C atoms were placed in calculated positions and treated as riding on their parent atoms, with C—H = 0.93 / 0.97 Å (aromatic / methylene) and with *U*_iso_(H) = 1.2*U*_eq_(C). Two O atoms of the nitrate anion were disordered over two positions with site occupancy of 0.58 for O1 and O2 atoms, and 0.42 for O1' and O2' atoms, respectively. The *SHELXL* 'DFIX' instruction was used to restrain the N—O bond distances of the disordered nitrate anion to close to 1.22 Å .

### Crystal data

**Table:**

[Cd(NO_3_)_2_(C_12_H_12_N_6_)_2_]	*Z* = 1
*M**_r_* = 716.97	*F*(000) = 362
Triclinic, *P*1	*D*_x_ = 1.617 Mg m^−^^3^
Hall symbol: -P 1	Mo *K*α radiation, λ = 0.71073 Å
*a* = 8.0412 (16) Å	Cell parameters from 7192 reflections
*b* = 8.7303 (17) Å	θ = 3.0–27.5°
*c* = 11.598 (2) Å	µ = 0.81 mm^−^^1^
α = 105.12 (3)°	*T* = 293 K
β = 90.20 (3)°	Block, colourless
γ = 109.71 (3)°	0.41 × 0.28 × 0.15 mm
*V* = 736.2 (2) Å^3^	

### Data collection

**Table:**

Rigaku R-AXIS RAPID diffractometer	3346 independent reflections
Radiation source: fine-focus sealed tube	3216 reflections with *I* > 2σ(*I*)
Graphite monochromator	*R*_int_ = 0.020
ω scan	θ_max_ = 27.5°, θ_min_ = 3.0°
Absorption correction: multi-scan (*ABSCOR*; Higashi, 1995)	*h* = −10→10
*T*_min_ = 0.734, *T*_max_ = 0.886	*k* = −11→10
7285 measured reflections	*l* = −15→15

### Refinement

**Table:**

Refinement on *F*^2^	Primary atom site location: structure-invariant direct methods
Least-squares matrix: full	Secondary atom site location: difference Fourier map
*R*[*F*^2^ > 2σ(*F*^2^)] = 0.030	Hydrogen site location: inferred from neighbouring sites
*wR*(*F*^2^) = 0.080	H-atom parameters constrained
*S* = 1.03	*w* = 1/[σ^2^(*F*_o_^2^) + (0.0539*P*)^2^ + 0.1306*P*] where *P* = (*F*_o_^2^ + 2*F*_c_^2^)/3
3346 reflections	(Δ/σ)_max_ < 0.001
224 parameters	Δρ_max_ = 0.77 e Å^−^^3^
3 restraints	Δρ_min_ = −0.68 e Å^−^^3^

### Special details

**Table:**

Geometry. All esds (except the esd in the dihedral angle between two l.s. planes) are estimated using the full covariance matrix. The cell esds are taken into account individually in the estimation of esds in distances, angles and torsion angles; correlations between esds in cell parameters are only used when they are defined by crystal symmetry. An approximate (isotropic) treatment of cell esds is used for estimating esds involving l.s. planes.
Refinement. Refinement of F^2^ against ALL reflections. The weighted R-factor wR and goodness of fit S are based on F^2^, conventional R-factors R are based on F, with F set to zero for negative F^2^. The threshold expression of F^2^ > 2sigma(F^2^) is used only for calculating R-factors(gt) etc. and is not relevant to the choice of reflections for refinement. R-factors based on F^2^ are statistically about twice as large as those based on F, and R- factors based on ALL data will be even larger.

### Fractional atomic coordinates and isotropic or equivalent isotropic displacement parameters (Å^2^)

**Table:**

	*x*	*y*	*z*	*U*_iso_*/*U*_eq_	Occ. (<1)
C1	0.5033 (3)	0.3521 (3)	0.2034 (2)	0.0506 (5)	
H1	0.4228	0.4026	0.1901	0.061*	
C2	0.6741 (3)	0.2753 (3)	0.29299 (19)	0.0456 (5)	
H2	0.7392	0.2575	0.3515	0.055*	
C3	0.7674 (4)	0.1037 (3)	0.1121 (2)	0.0503 (5)	
H3A	0.6804	−0.0067	0.0726	0.060*	
H3B	0.8445	0.0892	0.1697	0.060*	
C4	0.8758 (3)	0.1744 (3)	0.0203 (2)	0.0493 (5)	
C5	1.0410 (4)	0.3011 (5)	0.0530 (3)	0.0798 (10)	
H5	1.0883	0.3424	0.1329	0.096*	
C6	1.1364 (5)	0.3666 (6)	−0.0340 (4)	0.0995 (13)	
H6	1.2456	0.4541	−0.0117	0.119*	
C7	1.0688 (4)	0.3017 (5)	−0.1530 (3)	0.0782 (9)	
H7	1.1344	0.3438	−0.2109	0.094*	
C8	0.9050 (4)	0.1752 (3)	−0.1870 (2)	0.0524 (5)	
C9	0.8091 (3)	0.1123 (3)	−0.0998 (2)	0.0461 (5)	
H9	0.6983	0.0272	−0.1223	0.055*	
C10	0.8280 (4)	0.1018 (4)	−0.3175 (2)	0.0618 (7)	
H10A	0.9232	0.1280	−0.3682	0.074*	
H10B	0.7764	−0.0205	−0.3350	0.074*	
C11	0.7118 (3)	0.2961 (3)	−0.3915 (2)	0.0474 (5)	
H11	0.8197	0.3670	−0.4069	0.057*	
C12	0.4422 (4)	0.1801 (4)	−0.3748 (3)	0.0628 (6)	
H12	0.3203	0.1568	−0.3780	0.075*	
Cd1	0.5000	0.5000	0.5000	0.04272 (9)	
N1	0.5669 (3)	0.3636 (3)	0.31476 (17)	0.0487 (4)	
N2	0.5646 (3)	0.2640 (3)	0.11613 (18)	0.0532 (5)	
N3	0.6753 (2)	0.2162 (2)	0.17601 (16)	0.0421 (4)	
N4	0.5558 (3)	0.3100 (2)	−0.41081 (17)	0.0476 (4)	
N5	0.5189 (4)	0.0906 (3)	−0.3351 (3)	0.0695 (6)	
N6	0.6919 (3)	0.1667 (2)	−0.34697 (17)	0.0494 (4)	
N7	0.9115 (2)	0.7301 (3)	0.5706 (2)	0.0529 (5)	
O1	0.8078 (6)	0.6800 (8)	0.4850 (4)	0.093 (2)	0.575 (8)
O2	0.8601 (8)	0.6969 (6)	0.6706 (4)	0.0892 (18)	0.575 (8)
O1'	0.9178 (14)	0.5978 (8)	0.4970 (11)	0.166 (7)	0.425 (8)
O2'	0.7681 (6)	0.6944 (7)	0.6029 (8)	0.087 (3)	0.425 (8)
O3	1.0506 (2)	0.8468 (2)	0.58725 (18)	0.0585 (4)	

### Atomic displacement parameters (Å^2^)

**Table:**

	*U*^11^	*U*^22^	*U*^33^	*U*^12^	*U*^13^	*U*^23^
C1	0.0476 (12)	0.0654 (14)	0.0474 (11)	0.0266 (10)	0.0043 (9)	0.0209 (10)
C2	0.0513 (12)	0.0516 (11)	0.0396 (10)	0.0235 (9)	0.0035 (8)	0.0147 (9)
C3	0.0635 (14)	0.0542 (12)	0.0446 (11)	0.0304 (11)	0.0151 (10)	0.0196 (10)
C4	0.0494 (12)	0.0616 (13)	0.0489 (11)	0.0288 (10)	0.0145 (9)	0.0226 (10)
C5	0.0503 (15)	0.115 (3)	0.0644 (16)	0.0128 (16)	0.0023 (12)	0.0295 (17)
C6	0.0533 (18)	0.134 (3)	0.095 (3)	−0.0005 (19)	0.0142 (17)	0.049 (2)
C7	0.0623 (18)	0.110 (2)	0.079 (2)	0.0308 (17)	0.0312 (15)	0.0531 (19)
C8	0.0636 (14)	0.0671 (14)	0.0520 (12)	0.0444 (12)	0.0224 (10)	0.0301 (11)
C9	0.0516 (12)	0.0500 (11)	0.0467 (11)	0.0259 (9)	0.0130 (9)	0.0194 (9)
C10	0.093 (2)	0.0764 (16)	0.0493 (12)	0.0617 (16)	0.0242 (13)	0.0296 (12)
C11	0.0579 (13)	0.0468 (11)	0.0476 (11)	0.0249 (9)	0.0122 (9)	0.0212 (9)
C12	0.0568 (15)	0.0597 (14)	0.0770 (17)	0.0169 (11)	0.0113 (13)	0.0320 (13)
Cd1	0.05123 (15)	0.04842 (14)	0.03877 (13)	0.02770 (10)	0.00625 (8)	0.01561 (9)
N1	0.0545 (11)	0.0579 (11)	0.0424 (9)	0.0288 (9)	0.0050 (8)	0.0164 (8)
N2	0.0510 (11)	0.0723 (13)	0.0429 (9)	0.0262 (9)	0.0031 (8)	0.0209 (9)
N3	0.0433 (9)	0.0485 (9)	0.0392 (8)	0.0179 (7)	0.0077 (7)	0.0173 (7)
N4	0.0564 (11)	0.0451 (9)	0.0490 (10)	0.0229 (8)	0.0085 (8)	0.0191 (8)
N5	0.0727 (15)	0.0610 (13)	0.0887 (17)	0.0231 (11)	0.0196 (13)	0.0442 (13)
N6	0.0685 (13)	0.0499 (10)	0.0450 (9)	0.0330 (9)	0.0155 (9)	0.0219 (8)
N7	0.0389 (10)	0.0562 (11)	0.0699 (13)	0.0168 (8)	0.0083 (9)	0.0280 (10)
O1	0.057 (2)	0.124 (4)	0.078 (3)	0.010 (2)	−0.025 (2)	0.024 (3)
O2	0.077 (3)	0.105 (3)	0.076 (3)	0.004 (3)	0.016 (2)	0.047 (2)
O1'	0.127 (9)	0.108 (6)	0.159 (8)	−0.046 (6)	0.067 (7)	−0.023 (6)
O2'	0.043 (3)	0.076 (3)	0.115 (6)	0.009 (2)	0.028 (3)	−0.003 (3)
O3	0.0391 (8)	0.0582 (9)	0.0754 (12)	0.0123 (7)	0.0042 (8)	0.0201 (9)

### Geometric parameters (Å, º)

**Table:**

C1—N2	1.309 (3)	C10—H10B	0.9700
C1—N1	1.353 (3)	C11—N6	1.323 (3)
C1—H1	0.9300	C11—N4	1.324 (3)
C2—N3	1.321 (3)	C11—H11	0.9300
C2—N1	1.323 (3)	C12—N5	1.309 (4)
C2—H2	0.9300	C12—N4	1.358 (3)
C3—N3	1.475 (3)	C12—H12	0.9300
C3—C4	1.504 (3)	Cd1—O2'^i^	2.326 (6)
C3—H3A	0.9700	Cd1—O2'	2.326 (6)
C3—H3B	0.9700	Cd1—N1	2.330 (2)
C4—C5	1.385 (4)	Cd1—N4^ii^	2.3278 (19)
C4—C9	1.388 (3)	Cd1—N4^iii^	2.3278 (19)
C5—C6	1.394 (5)	Cd1—N1^i^	2.330 (2)
C5—H5	0.9300	Cd1—O1	2.479 (5)
C6—C7	1.378 (5)	Cd1—O1^i^	2.479 (5)
C6—H6	0.9300	N2—N3	1.359 (3)
C7—C8	1.378 (5)	N4—Cd1^iv^	2.3278 (19)
C7—H7	0.9300	N5—N6	1.349 (3)
C8—C9	1.388 (3)	N7—O2'	1.179 (4)
C8—C10	1.518 (4)	N7—O1	1.180 (3)
C9—H9	0.9300	N7—O3	1.206 (3)
C10—N6	1.468 (3)	N7—O1'	1.260 (5)
C10—H10A	0.9700	N7—O2	1.304 (5)
			
N2—C1—N1	114.4 (2)	O2'^i^—Cd1—N1	73.58 (14)
N2—C1—H1	122.8	O2'—Cd1—N1	106.42 (14)
N1—C1—H1	122.8	N4^ii^—Cd1—N1	88.62 (7)
N3—C2—N1	110.0 (2)	N4^iii^—Cd1—N1	91.38 (7)
N3—C2—H2	125.0	O2'^i^—Cd1—N1^i^	106.42 (14)
N1—C2—H2	125.0	O2'—Cd1—N1^i^	73.58 (14)
N3—C3—C4	111.58 (19)	N4^ii^—Cd1—N1^i^	91.38 (7)
N3—C3—H3A	109.3	N4^iii^—Cd1—N1^i^	88.62 (7)
C4—C3—H3A	109.3	N1—Cd1—N1^i^	180.0
N3—C3—H3B	109.3	O2'^i^—Cd1—O1	146.62 (19)
C4—C3—H3B	109.3	O2'—Cd1—O1	33.38 (19)
H3A—C3—H3B	108.0	N4^ii^—Cd1—O1	79.67 (14)
C5—C4—C9	119.1 (2)	N4^iii^—Cd1—O1	100.33 (14)
C5—C4—C3	121.4 (2)	N1—Cd1—O1	76.10 (15)
C9—C4—C3	119.5 (2)	N1^i^—Cd1—O1	103.90 (15)
C4—C5—C6	120.0 (3)	O2'^i^—Cd1—O1^i^	33.38 (19)
C4—C5—H5	120.0	O2'—Cd1—O1^i^	146.62 (19)
C6—C5—H5	120.0	N4^ii^—Cd1—O1^i^	100.33 (14)
C7—C6—C5	120.0 (3)	N4^iii^—Cd1—O1^i^	79.67 (14)
C7—C6—H6	120.0	N1—Cd1—O1^i^	103.90 (15)
C5—C6—H6	120.0	N1^i^—Cd1—O1^i^	76.10 (15)
C8—C7—C6	120.7 (3)	O1—Cd1—O1^i^	180.0
C8—C7—H7	119.7	C2—N1—C1	103.0 (2)
C6—C7—H7	119.7	C2—N1—Cd1	128.22 (15)
C7—C8—C9	119.1 (3)	C1—N1—Cd1	128.74 (16)
C7—C8—C10	121.6 (2)	C1—N2—N3	102.59 (18)
C9—C8—C10	119.3 (3)	C2—N3—N2	109.94 (19)
C4—C9—C8	121.1 (2)	C2—N3—C3	128.28 (19)
C4—C9—H9	119.4	N2—N3—C3	121.62 (18)
C8—C9—H9	119.4	C11—N4—C12	102.7 (2)
N6—C10—C8	113.12 (19)	C11—N4—Cd1^iv^	126.68 (16)
N6—C10—H10A	109.0	C12—N4—Cd1^iv^	130.47 (18)
C8—C10—H10A	109.0	C12—N5—N6	102.9 (2)
N6—C10—H10B	109.0	C11—N6—N5	110.0 (2)
C8—C10—H10B	109.0	C11—N6—C10	128.3 (2)
H10A—C10—H10B	107.8	N5—N6—C10	121.7 (2)
N6—C11—N4	110.1 (2)	O2'—N7—O1	72.1 (5)
N6—C11—H11	124.9	O2'—N7—O3	141.1 (4)
N4—C11—H11	124.9	O1—N7—O3	125.6 (3)
N5—C12—N4	114.3 (3)	O2'—N7—O1'	106.5 (5)
N5—C12—H12	122.9	O1—N7—O1'	66.5 (7)
N4—C12—H12	122.9	O3—N7—O1'	112.4 (4)
O2'^i^—Cd1—O2'	180.000 (1)	O2'—N7—O2	50.7 (4)
O2'^i^—Cd1—N4^ii^	85.9 (2)	O1—N7—O2	119.7 (4)
O2'—Cd1—N4^ii^	94.1 (2)	O3—N7—O2	111.6 (3)
O2'^i^—Cd1—N4^iii^	94.1 (2)	O1'—N7—O2	109.5 (6)
O2'—Cd1—N4^iii^	85.9 (2)	N7—O1—Cd1	118.1 (3)
N4^ii^—Cd1—N4^iii^	180.0	N7—O2'—Cd1	129.6 (5)

Symmetry codes: (i) −*x*+1, −*y*+1, −*z*+1; (ii) −*x*+1, −*y*+1, −*z*; (iii) *x*, *y*, *z*+1; (iv) *x*, *y*, *z*−1.
